# Reusing wastes from olive pomace in oil production chain: How membrane technology can rescue nutrients from wastewaters

**DOI:** 10.1016/j.heliyon.2025.e42653

**Published:** 2025-02-12

**Authors:** Alfredo Cassano, Pietro Calandra, Paolino Caputo, Rosanna Morelli, Carmela Conidi, Pietro Argurio, Cesare Oliviero Rossi

**Affiliations:** a National Research Council - Institute on Membrane Technology (CNR-ITM), c/o University of Calabria, Via P. Bucci, 17C, 87036, Rende, CS, Italy; b National Research Council, CNR-ISMN, Strada provinciale 35D n.9 - 00010, Montelibretti, RM, Italy; c University of Calabria – Department of Chemistry and Chemical Technologies, Via P. Bucci Cubo 14D, 87036, Rende, CS, Italy; d Department of Environmental & Chemical Engineering, University of Calabria, Via P. Bucci, 44/A, I-87036, Rende, Italy

**Keywords:** Pomace oil, Wastes, Circular economy, Wastewaters

## Abstract

In olive oil production, once high-grade oils (extra virgin olive oil and virgin olive oil) are produced by milling procedure, further oils are then recovered by different methods. The lowest grade is pomace oil, recovered usually by solvent techniques, from oil pomace. This step is important for the oil recovery, but environmental issue need to be faced, since the stocking and treatment of pomace oil causes emissions into the atmosphere with fumes and smells. The present work was aimed at evaluating the performance of nanofiltration (NF) and reverse osmosis (RO) processes in the treatment of wastewaters coming from the condensation of steam emissions in a pomace oil factory. This approach allowed to reduce the volume of condensed wastewaters producing two valuable streams: fresh water, as permeate stream, and a nutrient-rich solution, as retentate stream. The experimental results indicated a better performance of the RO membrane with a progressive increase of total organic carbon (TOC), conductivity and total polyphenols in the retentate of the process. This solution can be further dehydrated and reused as organic fertilizer. The purified water, produced as permeate, can be re-used within the plant itself (i.e. water replenishment of evaporative towers or as boiler feed after degassing) or in other processes (i.e. irrigation, machinery washing, etc.).

## 1 Introduction

Historically, olive pomace, a by-product of virgin olive milling process, is treated in pomace factories to extract pomace oil. This takes place on a seasonal basis, accompanying virgin oil seasonal production, and allows for the recovery of a certain amount of oil.

The extraction process of olive oil from olives is quite complex but typically follows a common trend which includes several steps such as collection, grading, washing, crushing, malaxing, separation and centrifugation. Six different types of oils with different organoleptic characteristics can be produced through specific modifications of such process, as defined by the European Commission regulation (Art. 118 No 1234/2007) and the International Olive Council (IOC).

The extraction of olive oil is accompanied by the production of substantial solid and liquid waste in the form of olive pomace and olive mill wastewater, respectively. On average, 35–40 kg of olive pomace are released per 100 kg of olives and the estimated production in the European Union Countries is more than 4.0 ⋅ 10^6^ m^3^/yr [1]. Olive pomace is typically constituted by water, olive skin, olive pulp, olive kernels and oil. The commercial value depends on its oil content typically ranging from 4 to 15 g per 100 g of dry matter [2].

Basically, the recovery of olive pomace oil from olive pomace is achieved by the combined action of (i) drying and (ii) extraction of residual oil via solvent.

(i) The drying is carried out in a rotary dryer, often fed with spent pomace produced on-site. During evaporation process water carries organic volatile compounds. In a typical pomace factory, around 15,000 kg/h of pomace, with humidity around 45–50 % wt/wt, is dried. This means that about 7000 kg of steam water is produced hourly. This is itself an environmental issue, constituting actually a long-standing problem still hard to solve.
(ii) The extraction is based on the immersion of the pomace into hexane; in this process, hexane acts as a solvent solubilizing the oil present in the pomace. After loading the dried pomace into the extractor and its washing with solvent, a draining and evaporation is induced to remove solvent residues from the exhausted pomace until the exhausted pomace is obtained. This second step is another source of unwanted substances delivered to the environment.

The entire pomace oil production is therefore source of heavily polluted wastewaters and solid wastes: being considered harmful to the environment, it is often focused by the local communities.

To better understand the importance of these issues, it must be noted that the value and work of a pomace factory is fundamental in the overall olive oil supply chain. This reflects on the production grand scheme of an entire country, particularly for Italy which is one of the main producers of olive oil [3]. This turns up in a need to develop innovative technologies aiming at limiting emissions and reusing the water wasted during the production process: in this ambit, scientific community is expected to pay attention to face these problems providing feasible solutions.

Different treatment methods and techniques have been proposed for the management of the wastewater and solid waste generated by olive pomace oil [4].

Olive oil pomace is a very abundant by-product and it is also considered as a good potential feedstock for biodiesel production [[5], [6], [7]] or composting through the addition of bulking agents [8]. The possibility of obtaining bioactive compounds, such as hydroxytyrosol, in high yield from olive oil pomace has been also investigated [9,10]. Extracts rich in phenolic compounds from olive pomace have been obtained through pressurized liquid extraction [11] and solid-liquid extraction [12].

An integrated valorization scheme where polyphenols are previously recovered in a high-value liquid extract to facilitate the subsequent continuous anaerobic digestion of dephenolised two-phase olive pomace has been also recently proposed [13]. In this view olive oil pomace is considered as a source of high nutritional value compounds to produce new functional additives or ingredients [14]. Integrated approaches to improve the working capacity and contribute to lower the environmental impact during all the process of olive oil production have been also proposed [15].

The complete removal of polycyclic aromatic hydrocarbons during pomace oil process and the utilization of distillates are important areas for research and development [16,17].

This study is pioneering in that it provides a simple approach for the treatment of steam emissions produced in a pomace factory with a potential for scaling up. It is inspired by the observation, from preliminary chemical analyses, that such emissions are rich in nitrogen and other nutritious. So, being a source of water, energy and organic substances, such wastes can be utilized, within a logic of circular economy, where a waste from a process can be added value ingredient to be used in another process, in the agricultural sector itself. Specifically, the investigated process was aimed at evaluating the performance of both NF and RO processes in the treatment of the aqueous condensate obtained from such emissions in order to reduce its volume with the production of fresh water, as permeate, reusable in the pomace factory as process water of for irrigation purposes and a concentrated solution (retentate), rich in organic compounds, of interest as source of micro- and macro-nutrients for fertilizing farmlands or related purposes.

The possibility of recovering emissions strongly points towards the direction of a circular economy with the dual advantage of not emitting vapors into the environment that could constitute disturbances (odors) to the local population while also obtaining reusable by-products in the supply chain.

## 2 Materials and methods

### 2.1 Feed solution

Condensed wastewaters were supplied by Olearia SOD located in Tarsia (Cosenza, Italy). These waters have been obtained simply by condensation of the final emissions of the steam of the plant. It is worthy to remark that the steam is pretreated by scrubbers in order to remove solid organic species from the steam.

### 2.2 Experimental set-up

Experiments were performed by using a NF/RO laboratory bench plant consisting of a control panel, a stainless steel cylindrical jacketed feed tank with a capacity of 5L, a feed plunger pump with belt drive (Cat Pumps, Milano-Italy, Model 3CP1221), two pressure gauges (Wika, Lawrenceville, GA, USA) (max pressure 100 bar, absolute error 1 bar), a digital flow meter (SM6000, IFM Electronic GmbH, Hamburg, Germany), a thermometer placed inside the feed tank and a cylindrical housing able to accommodate a 12 × 1.85 inches spiral-wound membrane module. The adjustment of operating pressure and feed flowrate was done by simultaneously pump rotation control through a frequency inverter and a needle valve. The operating temperature was controlled by circulating either a heating fluid or a coolant through the tank jacket.

For RO experiments the plant was equipped with a polyamide membrane module (SC1812) having a membrane surface area of 0.32 m^2^ and a NaCl rejection of 99.5 %. A polyamide composite membrane module (DK1812) with a molecular weight cut-off (MWCO) of 150–300 Da, a membrane surface area of 0.32 m^2^ and a MgSO_4_ rejection of 98 % was used for NF experiments. Both commercial membrane modules, in spiral-wound configuration, were supplied by GE Water & Process Technologies (Minnetonka, MN, USA).

RO experiments were performed according to the batch concentration configuration (in which the permeate is collected separately while the retentate is recycled back to the feed tank) under selected operating conditions (transmembrane pressure, 20 bar; feed flowrate, 2.75 L/min; temperature, 25 ± 1 °C), up to a volume concentration ratio (VCR) of 7.0. NF experiments were performed at a TMP (Trans Membrane Pressure) of 10 bar, a feed flowrate of 7.3 L/min and a temperature of 25 ± 1 °C up to a VCR of 3.9.

Volume concentration ratio was estimated according to the following equation:

(1) $$VCR=\frac{V_{r}}{V_{f}}$$

where *V*_*f*_ is the initial feed volume (L) and *V*_*r*_ is the retentate volume (L).

Permeate flux (J_p_), expressed as L/m^2^h, was calculated by measuring the volume of permeate collected in a certain time, according to the following equation:

(2) $$J_{p}=\frac{V_{p}}{A∙t}$$

where *V*_*p*_ (L) is the permeate volume at time *t* (h) and *A* is the membrane surface area (m^2^).

All experiments were conducted in triplicates. Permeate fluxes were reported as the average of the three different experiments ± the associated standard deviation. The data on means and standard deviations refer to measurements obtained from different membrane experiments, and not from replicate measurements from a single membrane experiment.

### 2.3 Analytical measurements

The total phenolic content was determined by the Folin-Ciocalteau method [18]. According to this method, 0.2 mL of the sample was combined with 1 mL of 10 % (v/v) Folin-Ciocalteau reagent (Sigma Aldrich) and 0.8 mL of a 7.5 % (w/v) sodium carbonate solution. The mixture was vortex and left to stand for 30 min at room temperature, the absorbance of the obtained solution was measured at wavelength of a 756 nm, by using a UV-160 UV–Visible recording spectrophotometer (Shimadzu Scientific Instruments, Inc., Japan). A calibration curve was done using gallic acid as a standard, and therefore TPC results are given as mg of gallic acid equivalents per liter (mg GAE/L).

Total organic carbon (TOC) was analyzed by a TOC analyzer (TOC-V CSN, Shimadzu, Kyoto, Japan). The sample is delivered to the combustion furnace, which is supplied with purified air. There, it undergoes combustion through heating to 680 °C with a platinum catalyst. It decomposes and is converted to carbon dioxide. The carbon dioxide generated is cooled and dehumidified, and then detected by the NDIR. The concentration of total carbon (TC) in the sample is obtained through comparison with a calibration curve formula. Furthermore, by subjecting the oxidized sample to the sparging process, the inorganic carbon (IC) in the sample is converted to carbon dioxide, and the IC concentration is obtained by detecting this with the NDIR. The TOC concentration is then calculated by subtracting the IC concentration from the obtained TC concentration. The concentrations of major cations and anions were determined by high performance liquid chromatography (HPLC, Dionex DX 1100, Sunnyvale, CA, USA).

Electrical conductivity was measured by using a digital conductivity meter (HI 2300 Microprocessor.

Conductivity, Hanna Instruments, Woonsocket, RI, USA).

The rejection (*R*) of selected membranes towards various components was calculated by using the following equation:

(3) $$R=\left(1-\frac{C_{p,i}}{C_{r,i}}\right)∙100$$

where, *C*_*p,i*_ represents the compound *i* concentration in the permeate, while *C*_*r,i*_ stands for its concentration in the retentate side.

All analytical measurements were conducted in triplicates and results were expressed as the average of the three measures ± the associated standard deviation. As for above, the data on means and standard deviations refer to measurements obtained from different membrane experiments, and not from replicate measurements from a single membrane experiment.

## 3 Results and discussion

### 3.1 Membrane productivity

**Fig. 1:**
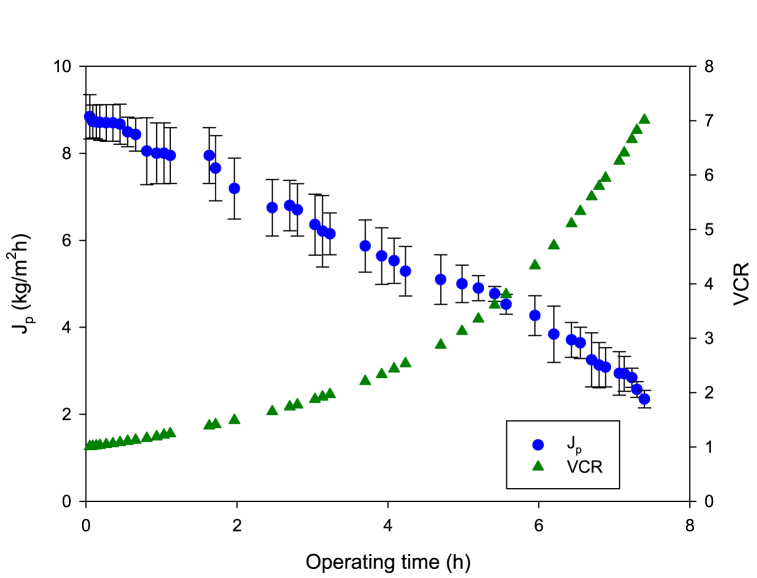
Reverse osmosis of condensed wastewaters. Time course of permeate flux and volume concentration ratio (VCR). Operating conditions: transmembrane pressure, 20 bar; feed flowrate, 2.75 L/min; temperature, 25 ± 1 °C.

Fig. 1 shows the time evolution of the permeate flux (*J*_*p*_) and volume concentration ratio (VCR) in the treatment of condensed wastewaters by RO according to the selected operating conditions. About 12.8 L of permeate were produced from 15 L of feeding wastewaters corresponding to a recovery factor of about 85 %, which is notable. The initial permeate flux was about 8.3 L/m^2^h, with a decreasing trend with time, reaching 1.1 L/m^2^h after 440 min. The permeate flux decline can be attributed to different factors, like the increase in the osmotic pressure during continuous concentration of the feed solution, as well as to membrane fouling and concentration polarization phenomena [19]. In particular, concentration polarization leads to a higher solute concentration at the membrane surface, which may cause higher local osmotic pressure close to the membrane surface, which means a flux decline when RO is performed at constant pressure [20].

Average permeate fluxes for the NF membrane, in the selected operating conditions, were of about 36 L/m^2^h.

**Fig. 2:**
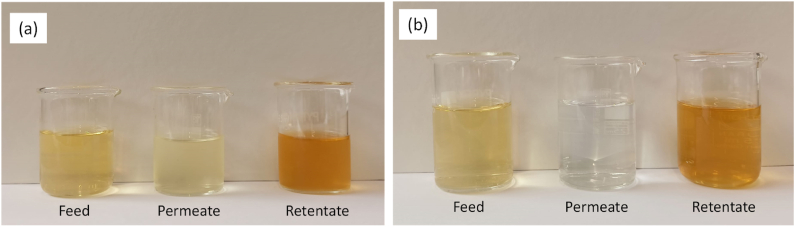
Samples produced in the treatment of condensed wastewater by nanofiltration (a) and reverse osmosis (b).

The samples produced in the treatment of condensed wastewater with both NF and RO membranes are illustrated in Fig. 2. As it can be seen even by visual inspection, the RO treatment produces a darker concentrated solution (retentate) and a clearer and uncolored solution (permeate) with respect the NF treatment.

**Fig. 3:**
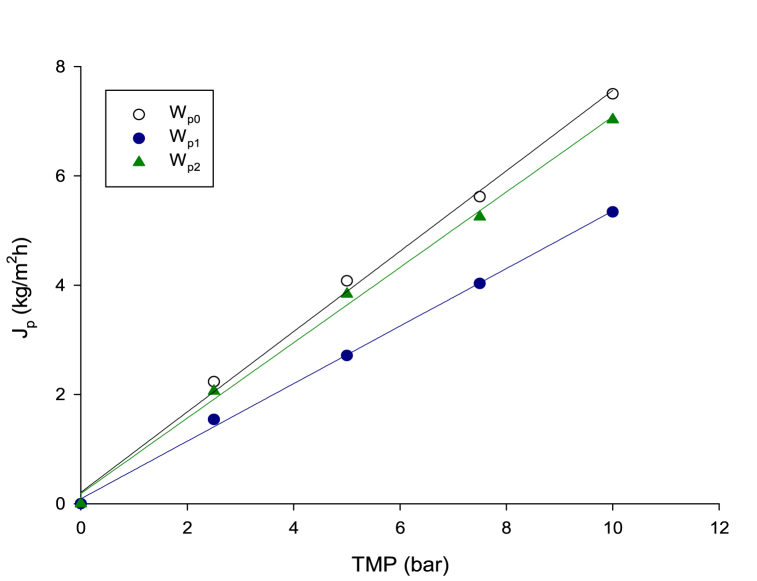
Water permeability of the RO membrane measured at 25 °C. W_p0_, original water permeability; W_p1_, water permeability after the treatment of condensed wastewater; W_p2_, water permeability after cleaning with water at 40 °C for 30 min.

In Fig. 3 the water permeability measured for the RO membrane before and after the treatment of condensed wastewater and after cleaning with water at 40 °C for 30 min is reported. Specifically, the original water permeability of the RO membrane of about 0.76 kg/m^2^ h bar decreased up to 0.54 kg/m^2^ h bar after the treatment of condensed wastewater. The water cleaning of the membrane at 40 °C for 30 min allowed to recover more than 96 % of the initial water permeability.

### 3.2 Analytical measurements

The analytical measurements related to the total phenolic content, the electrical conductivity and TC along with TOC and IC of feed, permeate and retentate samples from RO experiments are reported in Table 1.

**Table 1:** Analyses of total phenolic content, conductivity and total carbon (TC) in feed, permeate and retentate samples of the RO process (TOC, total organic carbon; IC, inorganic carbon).

	Total phenolic content (mg GAE/L)	Conductivity (mS/cm)	TC (mg/L)	TOC (mg/L)	IC (mg/L)
Feed	15.29 ± 0.55	5.88 ± 0.11	2784.8 ± 77	2783.0 ± 55.6	1.80 ± 0.02
Permeate	2.00 ± 0.07	0.956 ± 0.01	320.1 ± 6.4	318.8 ± 6.3	1.30 ± 0.03
Retentate	71.29 ± 2.57	30.5 ± 0.61	16502 ± 330	16497 ± 329	4.77 ± 0.09

The treated wastewater had a total phenolic content of 15.29 ± 0.55 mg GAE/L; TOC and electrical conductivity were of 2783.0 ± 55.6 mg/L and 5.88 ± 0.11 mS/cm, respectively. Based on these results, more than 87 % of phenolic compounds are rejected by the RO membrane, while the rejection for TOC is of about 88.5 %. On the other hand, when the condensed wastewater was treated by NF the rejection for phenolic compound and TOC was much lower (23.6 % and 30.8 %, respectively) indicating a greater diffusion of phenolic and organic compounds in the permeate stream (Table 2).

**Table 2:** Analyses of total phenolic content and conductivity in feed, permeate and retentate samples of the NF process.

	Total phenolic content (mg GAE/L)	Conductivity (mS/cm)	TOC (mg/L)
Feed	19.41 ± 0.38	3.63 ± 0.07	2820.5 ± 56.4
Permeate	14.82 ± 0.30	2.58 ± 0.05	1950.2 ± 40.6
Retentate	43.53 ± 0.87	5.86 ± 0.11	5117.9 ± 102.3

**Fig. 4:**
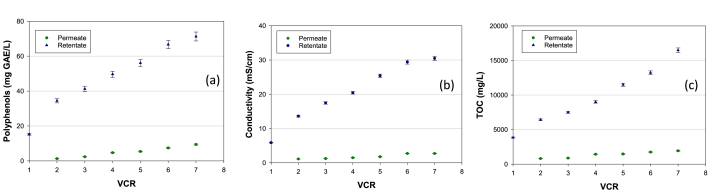
Total polyphenols concentration (a), conductivity (b) and TOC (c) in permeate and retentate streams of the RO process as a function of VCR.

Fig. 4 shows the content of polyphenols, the conductivity and the total organic carbon (TOC) measured in the permeate and retentate streams of the RO process as a function of VCR. All these parameters increase in the retentate stream along with the VCR of the RO process; on the other hand, they remain almost constant in the permeate, assessing at very low values (less than one order of magnitude lower than the feeding solution).

Analyses of specific anions and cations in feed, permeate and retentate samples, at the end of the RO process, are reported in Table 3. The corresponding rejection values are notable. They are: 98 % for Cl^−^, 94 % for NO_2_^−^, 96 % for Br^−^, 96 % for NO_3_^−^, 98.5 % for SO_4_^2−^, 99 % for Na^+^, 97 % for K^+^, 94 % for Ca^2+^ and 95 % for Mg^2+^, which are quite high. Similar rejection values were reported for Ca^2+^, Mg^2+^ and Na^+^ in the treatment flue-gas desulfurization wastewaters with commercial thin film composite polyamide RO membranes (SWC-2540 and ESPA-2540, from Hydranautics) in spiral-wound configuration [21].

**Table 3:** Concentration (expressed as mg/L) of anions and cations in the permeate and retentate samples of the RO process.

	Cl^−^	NO_2_^−^	Br^−^	NO_3_^−^	SO_4_^2-^	Na^+^	K^+^	Ca^2+^	Mg^2+^
Feed	45 ± 2	18.1 ± 0.9	15.8 ± 0.8	26.5 ± 1	140 ± 7	5.7 ± 0.3	142 ± 7	10.9 ± 0.5	1.70 ± 0.08
Permeate	5.8 ± 0.3	4.1 ± 0.2	4.1 ± 0.2	4.8 ± 0.2	11.2 ± 0.5	0.50 ± 0.02	23 ± 1	3.3 ± 0.1	0.405 ± 0.002
Retentate	260 ± 13	66 ± 3	99 ± 5	126 ± 6	755 ± 40	35 ± 2	670 ± 30	53 ± 3	8.7 ± 0.4

### 3.3 Challenge applications

All the results self-consistently indicate that the RO treatment of aqueous condensed wastewaters from steam emissions produced in the pomace factory is an ecofriendly and sustainable approach to mitigate environmental concerns associated to this industrial sector within a logic of circular economy. This allows for a better managing of the waste solutions, since being more concentrated, they can occupy much lower volumes. On the other hand, the production of clear water as RO permeate is another advantage of the process, since it can be re-used in the plant for thermal exchange.

**Fig. 5:**
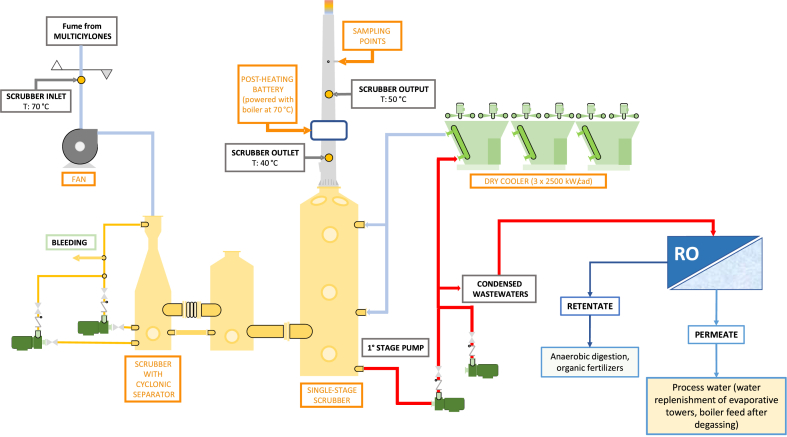
General flow-sheet of the treatment of olive pomace fumes integrated with the processing of condensed wastewaters by RO.

In the light of the experimental results, a general and integrated flow-sheet for the management of olive pomace fumes is depicted in Fig. 5. Condensed wastewaters concentrated by RO (retentate) can be submitted to an anaerobic digestion for the production of biogas. Another interesting application of retentate could be the development of organic fertilizers. In this ambit, in fact, it must be considered that washing water from olive pomace as well as the virgin pomace is already used as irrigation waters and fertilizers respectively. For example, in a country with a longstanding tradition in preparation and usage of olive-based products, like Italy, usage laws are clear in this respect [22]. Therefore, considering that our retentate is derived from a water assimilable to such kind of a product, we can rule out, in principle, ecological risks if a wise use of our retentate is made. In this framework, the optimized use can depend on the specific soil characteristics, as properties like pH and organic load [23]. In any case, as reported by this review, “it is generally reported that such wastewaters amendment causes minor long-term effects on soil microflora and that no evidence of any inhibitory effect on the growth of soil microorganisms was recorded”. Of course, each country is owed to eventually provide specific regulations on the basis of local problematics, if needed.

The RO permeate can be recycled in the pomace factory as process water, i.e. as water replenishment of evaporative towers or as boiler feed after degassing.

## 4 Conclusions

In the olive oil industry raw olive pomace, as by-product of the olive oil extraction, is processed into olive pomace oil with the production of unwanted emissions.

In this work, RO and NF membranes have been tested, for the first time, to treat the condensed wastewater produced from the steam emissions of a pomace factory. Average permeate fluxes of about 4.7 L/m^2^h and 36 L/m^2^h were obtained with selected polymeric RO and NF membranes at operating pressures of 20 and 10 bar, respectively.

The rejection of the RO membrane toward TOC and total phenolic content resulted of about 88.5 and 87 %, respectively. Anions (i.e. Cl^−^, NO_2_^−^, Br^−^, NO_3_^−^, SO_4_^2−^) and cations (i.e. Na^+^, K^+^, Ca^2+^, Mg^2+^) were rejected for more than 94 %. On the other hand, rejections for TOC and phenolic compounds measured in the NF process were of much lower that those observed for the RO process (23.6 % and 30.8 %, respectively) indicated a greater diffusion of organic and phenolic compounds through the NF membrane.

A progressive increment of electrical conductivity, TOC and total phenolic content was observed in the retentate stream of the RO process by increasing the volume concentration ratio of the process; on the other hand, these parameters remained quite constant on the permeate side.

The RO treatment produces two valuable fractions: (i) a retentate stream, i.e. a nutrient-rich solution, which can be used for organic fertilization or, alternatively, for the production of biogas through anaerobic digestion; (ii) a permeate stream, depleted for most part of organic compounds and salts, which can be reused within the plant itself, in water replenishment of evaporative towers or as boiler feed after degassing.

Overall, the RO treatment of the condensed wastewaters is a promising approach to reduce the formation of smells and the production of pollutant wastewaters in pomace oil plants with undiscussed environmental and economic benefits associated to the potential reuse of both permeate and retentate streams.

As future perspectives, we suggest further investigations to identify the most suitable crops to be irrigated considering the chemical composition of the retentate streams in order to prevent potential ecological risks, which unavoidably must include legislative aspects according to the specific Country considered. Additionally, optimization of operating and fluid-dynamic conditions as well as of membrane cleaning procedures are other aspects which should be properly analyzed in order to proceed to a proper assessment of the operational costs or the frequency of membrane replacements.

## CRediT authorship contribution statement

**Alfredo Cassano:** Writing – original draft, Investigation, Data curation, Conceptualization. **Pietro Calandra:** Writing – original draft, Conceptualization. **Paolino Caputo:** Resources. **Rosanna Morelli:** Methodology, Investigation. **Carmela Conidi:** Methodology, Investigation, Data curation. **Pietro Argurio:** Investigation, Data curation. **Cesare Oliviero Rossi:** Supervision, Conceptualization.

## Data availability statement

Data will be made available on request.

## Declaration of competing interest

The authors declare that they have no known competing financial interests or personal relationships that could have appeared to influence the work reported in this paper.
